# Accurate Calibration and Uncertainty Estimation of the Normal Spring Constant of Various AFM Cantilevers

**DOI:** 10.3390/s150305865

**Published:** 2015-03-10

**Authors:** Yunpeng Song, Sen Wu, Linyan Xu, Xing Fu

**Affiliations:** State Key Laboratory of Precision Measuring Technology and Instruments, Tianjin University, Tianjin 300072, China; E-Mails: ypsong@tju.edu.cn (Y.S.); senwu@tju.edu.cn (S.W.); xingfu@tju.edu.cn (X.F)

**Keywords:** AFM cantilever, normal spring constant, calibration, balance, uncertainty estimation

## Abstract

Measurement of force on a micro- or nano-Newton scale is important when exploring the mechanical properties of materials in the biophysics and nanomechanical fields. The atomic force microscope (AFM) is widely used in microforce measurement. The cantilever probe works as an AFM force sensor, and the spring constant of the cantilever is of great significance to the accuracy of the measurement results. This paper presents a normal spring constant calibration method with the combined use of an electromagnetic balance and a homemade AFM head. When the cantilever presses the balance, its deflection is detected through an optical lever integrated in the AFM head. Meanwhile, the corresponding bending force is recorded by the balance. Then the spring constant can be simply calculated using Hooke’s law. During the calibration, a feedback loop is applied to control the deflection of the cantilever. Errors that may affect the stability of the cantilever could be compensated rapidly. Five types of commercial cantilevers with different shapes, stiffness, and operating modes were chosen to evaluate the performance of our system. Based on the uncertainty analysis, the expanded relative standard uncertainties of the normal spring constant of most measured cantilevers are believed to be better than 2%.

## 1. Introduction

The Atomic Force Microscope (AFM) is a typical surface imaging instrument for micro- or nano-scale specimens. More recently, researchers have adopted it to measure force down to picoNewton (pN) scale in biophysics and nanomechanical fields [1,2]. Such applications need to measure or control the force between the force sensor (the AFM cantilever probe) and the sample surface. The force measurements rely on cantilevers with a known spring constant. However, as current micromachining process cannot precisely control the dimensions (especially the thickness) and material properties of each cantilever, probe manufacturers usually give a nominal spring constant with a wide range for each cantilever model. Thus, in precision force metrology, the spring constant of each cantilever must be calibrated properly [3].

Over the last 20 years, many techniques have been developed to calibrate the spring constant of AFM cantilevers. In principle, these techniques can be classified into three main categories: dimensional methods, dynamic methods and static bending methods [4,5,6]. In the dimensional methods, the geometrical dimensions and material properties of the cantilever are used to calculate the spring constant [7,8,9]. These methods are applicable for rectangular shaped cantilevers with a simple equation, but for complex geometry such as triangular shaped or trapezoid shaped cantilevers, finite element analysis is often applied to obtain a more credible result. The uncertainty of dimensional methods ranges from 10% to 20%, and the main sources of the uncertainty are the measured thickness and the unreliable Young’s modulus [6]. In dynamic methods, the spring constant is mainly measured based on the cantilever’s resonant response. The most widely used methods include the Cleverland method [10,11], Sader method [12,13,14], thermal tune method [15,16,17] and laser Doppler vibrometry method [18,19]. Some of the methods, such as the thermal tune method, are easy to implement and available in newly designed commercial AFM systems, but dynamic methods are not suitable for all kinds of cantilevers and the relative uncertainties of these methods are typically 10%~30%. The static bending methods are the most direct methods in determining the spring constant. Small force facilities or reference devices with known mechanical properties are used to apply a constant force to the tip of the cantilever. Then with the measurement of the corresponding deflection of the cantilever, the spring constant can be calculated based on Hooke’s law. Typical static methods include the reference cantilever method [20,21], the nanoindentation method [22] and the balance method [23,24,25,26,27]. Recently, researchers have focused on the topic of SI traceability in nanoforce metrology, so the balance method which could calibrate the spring constant in a SI-traceable manner has received more and more attention. Among the various balance methods, a standard uncertainty of less than 1% was achieved with the nanoforce calibrator (NFC) presented by Kim *et al.* [24]. However, due to the fact that prior research using balance methods did not measure the deflection of the cantilever directly, some errors caused by the uncertain displacement of the cantilevers may affect the accuracy of the calibration results.

In our recent study, a cantilever calibration facility that combines an electromagnetic balance and a home-made AFM head has been set up [26,27]. Different from the previous balance method, our calibration facility can directly measure the deflection of the cantilever and the corresponding bending force. With this system, we can determine the normal spring constant of almost all kinds of cantilever probes, regardless of their shapes and stiffness. Five kinds of commercial AFM cantilevers with different applications and nominal spring constant ranges were calibrated, and the uncertainties of the measurement results were estimated systematically.

## 2. Calibration Facility and Method

### 2.1. Facility Setup

**Figure 1:**
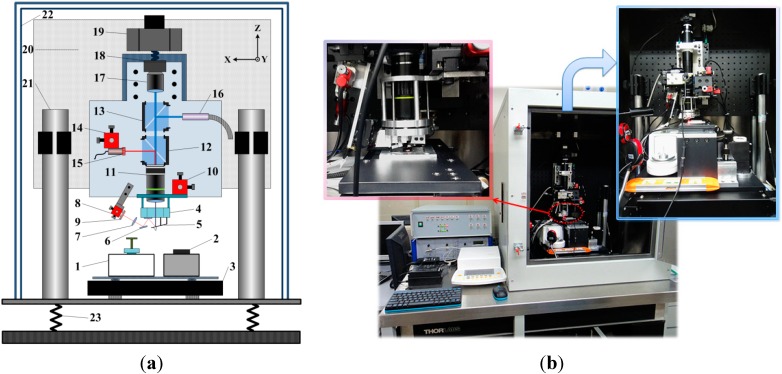
Schematic (**a**) and photos (**b**) of the calibration facility. The numbered components in (**a**) are: (1) Precision electromagnetic balance; (2) Nano positioning stage; (3) X-Y axis motor stage; (4) Piezo scanner; (5) Cantilever holder; (6) Reflector; (7) Lens; (8, 10, 14) Ultra-precision 3D motor stages; (9) PSD; (11) Objective; (12, 13) Beam splitters; (15) Laser diode; (16) Illuminator; (17) Tube lens; (18) CCD; (19) Z-axis motor stage; (20) Supporting breadboard; (21) Pillars; (22) Shielding case; (23) Optical vibration isolation table.

Figure 1 show a schematic and photos of the calibration setup. Our calibration facility consists of an optical microscope (11–13, 16–18), an optical lever system (5–7, 9, 11, 15), a precision electromagnetic balance (1), a piezo scanner (4), several positioning stages with micro- or nanometer resolution (2, 3, 8, 10, 14, 19) and some auxiliary control units. The whole system is mounted on an optical vibration isolation table (23) and enclosed in a shielding case (22) with a relatively constant temperature to reduce the interference from environmental thermal drift, mechanical vibrations, airflows and acoustic noises. The measured cantilever is fixed in a commercial cantilever holder (5) and mounted under the piezo scanner (4) with 6 μm working range and 2 nm resolution, so the measured spring constant is the effective spring constant of the cantilever when it is used in the corresponding AFM equipment. In our case, a cantilever holder from Dimension3100M (Digital Instrument, Plainview, NY, USA) is used, and the inclination angle of the cantilever relative to the horizontal plane is 12°.

**Figure 2:**
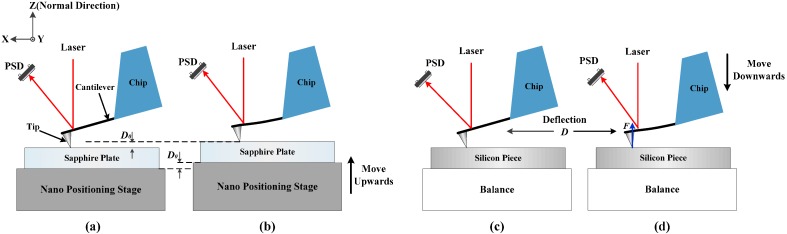
Schematic of the normal spring constant calibration principle. (**a**,**b**) are the process of calibration of the optical lever sensitivity. (**c**,**d**) are the process of force calibration.

The normal spring constant is defined as the ratio of the applied normal force *F* at the tip of the probe to the deflection of the cantilever in the normal direction at the tip position *D*, as shown in Figure 2d. An optical lever system is integrated in our calibration facility to measure the deflection of the cantilever. The laser beam (635 nm, 4.5 mW) is passed through the aperture in the center of the piezo scanner and focused on the back side of the cantilever. Then the cantilever reflects the laser beam to a dual-axis position sensitive detector (PSD) (9). An Ultra-precision 3D motor stage (step length resolution <30 nm, New Focus Corp., Santa Clara, CA, USA) (10) is applied to position the piezo scanner together with the attached cantilever under the microscope. Since the focused laser spot is fixed in the image field of the microscope, one may align the 3D motor stage (10) and make the laser beam focus on a proper position of the cantilever.

### 2.2. Calibration Method

Before the spring constant calibration, the sensitivity of the optical lever should be measured first. As shown in Figure 2a,b, a nanopositioning stage (P733.3CL, PI GmbH, Karlsruhe, Germany) with a sapphire plate fixed on top was used to push the tip upwards and generate a deflection of the measured cantilever. Then the optical lever detects the change of the cantilever deflection and shifts the laser spot on the PSD. As the deflection of the cantilever was quite small in our calibration, the PSD output signals and the deflection of the cantilever should conform to a linear relationship. Since the sapphire plate is approximated as rigid and the spring constant of the cantilever is much smaller than other structures in the system, the deflection of the free end of the measured cantilever is equal to the vertical movement (positive direction of Z-axis) of the nanopositioning stage. Then the optical lever sensitivity *S* can be defined as the ratio of the shift of the PSD output signal *U*_0_ to the vertical movement *D*_0_ of the nano positioning stage, as shown in Equation (1):

(1) $$S=\frac{U_{0}}{D_{0}}$$

For any cantilevers to be calibrated, the sensitivity of the optical lever must be measured properly and accurately.

After the calibration of the optical lever sensitivity, the deflection *D* of the cantilever in the following force calibration step can be calculated by the ratio of the increase of the PSD output signal *U* to the optical lever sensitivity *S*, as shown in Equation (2):

(2) $$D=\frac{U}{S}$$

Then the nanopositioning stage is moved away by an X-Y axis motor stage (3), and a precision electromagnetic balance (1) was placed under the cantilever instead. The balance used in our system is a compensating type precision electromagnetic balance (SE2, Sartorius GmbH, Göttingen, Germany) with 2.1 g capacity and 0.1 μg resolution. The added mass *m* measured by the balance can be converted into the corresponding force by multiplying by the local acceleration of gravity *g*, so the balance is able to measure a force in the range of approximate 0~21 mN with a resolution of better than 1 nN. The balance operates in a force-compensation method. When an unknown force is applied on the balance, an electromagnetic force generated by a coil and a permanent magnet will counteract the external force to maintain the weighting pan of the balance in its initial position.

A Z-axis motor stage (19) is used for coarse positioning of the cantilever in the *Z* direction. When the tip slightly touches the balance, the measured cantilever is pushed downwards (negative direction of the Z-axis) by the piezo scanner to press on the balance, as shown in Figure 2c,d. Then the contact force *F* and the deflection *D* of the cantilever are measured and recorded by the balance and the optical lever system synchronously. With this process, the normal spring constant of the cantilever *k* could be calculated using Equation (3):

(3) $$k=\frac{F}{D}=\frac{mg}{U/S}=\frac{mgS}{U}$$

## 3. Normal Spring Constant Calibration

### 3.1. Cantilevers

In order to investigate the performance of our calibration system, we chose five kinds of commercial AFM cantilevers with different shapes and application modes, and calibrated the normal spring constant of seven cantilevers (including three NSC15_F cantilevers from the same box). The nominal spring constants of these cantilevers ranged from 0.03 N·m^−1^ to 46 N·m^−1^. Table 1 lists the information of the cantilevers to be measured as provided by the respective manufacturer.

**Table 1:** The information of the cantilevers provided by the datasheets from the manufacturer.

Model	Application Mode	Shape	Dimension (μm)				Resonant Frequency (kHz)	Spring Constant *k* (N·m^−1^)	
			Length	Width	Thickness	Tip Height		Nominal	Range
**CSG01**	Contact	Rectangular	350 ± 5	30 ± 3	1.0 ± 0.5	14~16	4~17	0.03	0.003~0.130
**NSG01**	Tapping	Rectangular	125 ± 5	30 ± 3	2.0 ± 0.5	14~16	87~230	5.10	1.45~15.10
**NSC11**	Electrostatic Force	V-shape	200 ± 5	40 ± 3	2.0 ± 0.3	15~20	50~80	3.0	1.5~5.0
**MESP**	Magnetic Force	Rectangular	225 ± 25	28 ± 5	3.0 ± 0.5	10~15	50~100	2.8	1.0~5.0
**NSC15_F**	Force Spectroscopy	Rectangular	125 ± 5	35 ± 3	4.0 ± 0.5	20~25	265~400	46	20~75

### 3.2. Calibration Experiment

Two cantilevers (NSC11 and NSC15_F#1) were selected from the measured cantilevers to discuss the calibration experiments in detail. Before a calibration, the optical lever system should be adjusted carefully. Then the cantilever is put in the cantilever holder and mounted under the piezo scanner. With the precise adjustment of the ultra-precision 3D motor stages, the laser beam is focused on the back side of the cantilever and reflected to the PSD. As mentioned above, the calibration processes include two steps: the calibration of the optical lever sensitivity and the force calibration.

#### 3.2.1. Calibration of the Optical Lever Sensitivity

**Figure 3:**
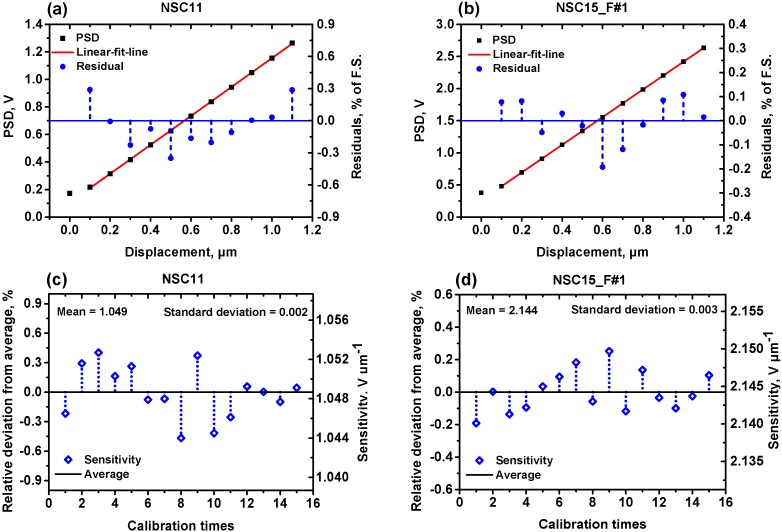
Optical lever sensitivity calibration curves of NSC11 and NSC15_F#1 cantilever. (**a**,**b**) are the voltage-displacement relationships; (**c**,**d**) are the scatters of the optical lever sensitivity obtained from 15 measurements.

In the first step, the measured cantilever was positioned several microns above the nanopositioning stage by the Z-axis motor stage. Then the nanopositioning stage is moved upwards (positive direction of Z-axis) to approach the cantilever. Once the sapphire slightly touched the tip of the probe, the rest of the sensitivity calibration sequences were automated. The nanopositioning stage moved in steps to bend the cantilever. In each step, the corresponding PSD output signals were recorded simultaneously. Figure 3a,b show the typical voltage-displacement curves of the cantilevers (NSC11 and NSC15_F#1) in the optical lever sensitivity calibration.

The first two points indicate that physical contact between the tip and the sapphire began from the second step. The measured cantilevers were pushed by the nanopositioning stage and bent 1 μm in 10 steps. Then the recorded data points except the first one were fitted using a least-square line. The calibration results presented a linear relationship between the deflection of the cantilever and the PSD output signals. For both cantilevers, the relative residual of each point was less than ±0.5%. The slope of the linear fit line was one measurement result of the sensitivity. Then the calibration was repeated 15 times, and the mean value was regarded as the sensitivity of the optical lever for the measured cantilever. As depicted in Figure 3c,d, the measurement results of the two cantilevers showed good repeatability with relative deviation of less than ±0.5% from the average. Results with small residuals and relative deviations were also achieved in the calibration of the other five cantilevers.

#### 3.2.2. Force Calibration

After the calibration of the optical lever sensitivity, the balance was placed under the cantilever to perform a force calibration. Then after the levelling and internal self-calibration of the balance, a 20 mm height aluminum column with a polished monocrystal silicon piece (500 μm thickness with no covering layers) on top was fixed on the weighting pan of the balance (made of stainless steel) to make a flat contact between the cantilever tip and the balance and reduce possible electromagnetic interference effects in the force measurement process. By using the Z-axis motor stage and the piezo scanner, the measured cantilever moved downwards (negative direction of Z-axis) to approach the balance. When the tip slightly touched the silicon piece, the piezo scanner extended in steps to bend the cantilever.

**Figure 4:**
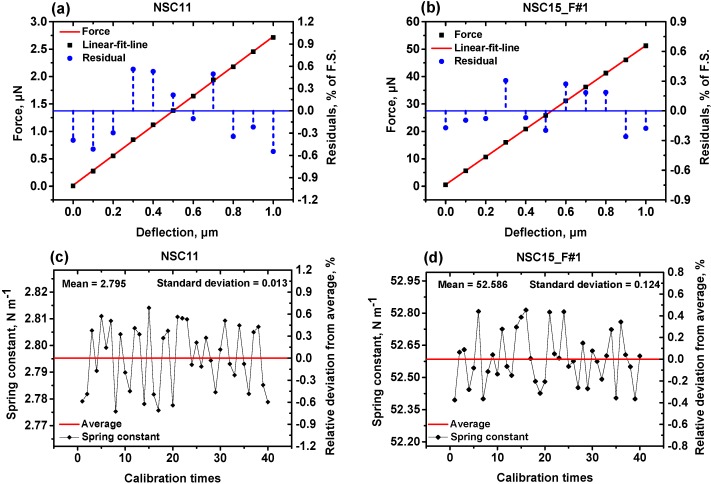
Force calibration curves of NSC11 and NSC15_F#1 cantilever. (**a**,**b**) are the force-deflection relationships; (**c**,**d**) are the scatters of measurement results.

During the force calibration, the deflection of the cantilever was servo-controlled by a PID controller. In the closed-loop system, the PSD output voltage was used as a feedback signal and compared with a setpoint value. The shift of the cantilever deflection changed the PSD output voltage. If the PSD output voltage was different from the setpoint value, the PID controller was triggered and used to adjust the driving voltage of the piezo scanner. Then the error of the deflection was compensated by the displacement of the piezo scanner. With this process, the deflection of the cantilever could be controlled in real-time and keep stable during the calibration. Errors which might influence the bending of the cantilever were totally compensated. In our force calibration, the deflection of the cantilever was increased in 10 equal steps the same as the calibration of the optical lever sensitivity. Figure 4a,b present the force-deflection curves of the same cantilevers (NSC11 and NSC15_F#1) mentioned above. The horizontal axis is obtained from Equation (2). The corresponding vertical axis is calculated by *F* = *mg*. A good linear relationship was found in each figure with small residuals of less than ±0.6%. The slope of the linear fit line was considered as one measurement result of the normal spring constant.

Then we repeated the force calibration 40 times in a period of time to check the measurement repeatability. The mean value was taken as the calibrated normal spring constant of the measured cantilever. As can be seen in Figure 4c,d, the calibration results have no obvious drifts, and the relative deviations from the average value were all less than 0.8%. Besides the cantilevers discussed above, we have calibrated five more cantilevers with the same method and procedures. The calibration results of the seven measured cantilevers are listed in Table 2. These cantilevers have different applications and nominal spring constants, but the relative standard deviations in the repeated measurement of all the calibration results were small. It means that our calibration facility is able to accurately calibrate the normal spring constant of a cantilever regardless of its shape, dimensions, and stiffness.

**Table 2:** Calibration results of different cantilevers.

Cantilever	Spring Constant *k* (N·m^−1^)		Relative Standard Deviation of Mean (%)
	Nominal	Calibrated (Mean)	
**NSC11**	3.0	2.795	0.074
**CSG01**	0.03	0.0273	0.093
**NSG01**	5.10	8.316	0.067
**MESP**	2.8	4.022	0.079
**NSC15_F#1**	46	52.586	0.036
**NSC15_F#2**	46	51.304	0.059
**NSC15_F#3**	46	44.035	0.075

## 4. Uncertainty Estimation

A mathematical model based on Equation (3) has been set up to complete the uncertainty estimation of the measurement results. In this model, four uncertainty sources: the balance added mass *m*, the acceleration of gravity *g*, the increase of the PSD output signal *U*, and the optical lever sensitivity *S* were considered. There are only product and divide forms in the input parameters, so the combined relative standard uncertainty of the normal spring constant can be described by Equation (4):

(4) $$u_{crel}(k)=\sqrt{u_{rel}^{2}(m)+u_{rel}^{2}(g)+u_{rel}^{2}(U)+u_{rel}^{2}(S)}$$

where *u*_rel_(*m*), *u*_rel_(*g*), *u*_rel_(*U*) and *u*_rel_(*S*) are the components of the relative standard uncertainty of the measured spring constant. The NSC15_F#1 cantilever mentioned above is chosen to discuss the uncertainty estimation in detail.

### 4.1. Uncertainty of the Added Mass, u_rel_(m)

The uncertainty of the balance added mass was combined with the uncertainties of the resolution, the reproducibility of the balance, and the measurement repeatability of added mass. According to the handbook and the calibration certification provided by the manufacturer, the resolution and the reproducibility of the precision balance are 0.1 μg and 0.25 μg, respectively. Assumed as uniform distribution, the uncertainty contributed by the resolution and the reproducibility of the balance could be estimated as $u_{1}(m)=0.1/2\sqrt{3}=0.00003\text{ mg}$ , $u_{2}(m)=0.00025\text{ mg}$ . The degrees of freedom (DOF) of the two components were:

(5) $$v_{1}(m)=v_{2}(m)=\frac{1}{2\times {\left(10\%\right)}^{2}}=50$$

In the force calibration, the experiment was repeated *n* = 40 times. The average and experimental standard deviation of the added mass were $\bar{m}=5.0042\text{ mg}$ and:

(6) $$s(m)=\sqrt{\frac{\sum_{i=1}^{n}{\left(m_{i}-\bar{m}\right)}^{2}}{n-1}}=0.01189\text{ mg}$$

The experimental standard deviation of the average value of 40 times measurement is calculated as:

(7) $$s(\bar{m})=s(m)/\sqrt{n}=0.01189/\sqrt{40}=0.00188\text{ mg}$$

So the uncertainty of the measurement repeatability of added mass was expressed as $u_{3}(m)=s(\bar{m})=0.00188\text{ mg}$ .The corresponding DOF was given by *v*_3_(*m*) = *n* − 1 = 39.

The standard uncertainty of added mass was determined by the uncertainty of three components:

(8) $$u(m)=\sqrt{u_{1}^{2}(m)+u_{2}^{2}(m)+u_{3}^{2}(m)}=\sqrt{0.00003^{2}+0.00025^{2}+0.00188^{2}}=0.00190\text{ mg}$$

The relative standard uncertainty and the degrees of freedom of the added mass were given by:

(9) $$u_{rel}(m)=\frac{u(m)}{m}=\frac{0.00190}{5.0042}=0.038\%,v_{eff}(m)=\frac{u^{4}(m)}{\sum_{i=1}^{3}\frac{u_{i}^{4}(m)}{v_{i}(m)}}=40.7$$

### 4.2. Uncertainty of the Acceleration of Gravity, u_rel_(g)

According to the data from Gravity Network Center of China (GNCC), the local acceleration of gravity is *g* = 9.8011 m·s^−2^, and the corresponding standard uncertainty is 0.0010 m·s^−2^. So the uncertainty of the acceleration of gravity would be:

(10) $$u_{rel}(g)=\frac{0.0010}{9.8011}=0.010\%,v_{eff}(g)=\frac{1}{2\times {\left(10\%\right)}^{2}}=50$$

### 4.3. Uncertainty of the Increase of PSD Output Signal, u_rel_(U)

In the force calibration, the deflection of the cantilever was controlled and adjusted by a closed loop system. The increase of PSD output signal *U* was assigned, it should equal to the setpoint signal. According to the experimental data, the uncertainty caused by the PSD output signal errors during the force calibration was smaller than 0.002%. It has negligible contribution to the combined uncertainty estimation results. With the feedback control loop, the uncertainty caused by the thermal drifts and the finite stiffness of the elements in the system did not need to be considered.

### 4.4. Uncertainty of the Optical Lever Sensitivity, u_rel_(S)

Equation (1) is the principal formula for the optical lever sensitivity *S*. It is the transparent-box model part of the mathematical model for uncertainty estimation. The laser spot shift on the cantilever *P* is also an uncertainty source for the optical lever sensitivity *S*, and should be introduced into Equation (1) as a correction factor (the black-box model part) to complete the mathematical model. The full mathematical model for the uncertainty estimation of the optical lever sensitivity *S* can be described by Equation (11):

(11) $$S=\frac{U_{0}}{D_{0}}P$$

According to Equation (11), the uncertainty of three components, the shift of the PSD output signal *U*_0_, the vertical displacement of the nanopositioning stage *D*_0_, and the laser spot shift on the cantilever *P* should be evaluated, respectively.

In the calibration of the optical lever sensitivity, *U*_0_ was measured *n* = 15 times with the same cantilever deflection. The expectation of *U*_0_ and its experimental standard deviation were $\bar{U_{0}}=2144.32\text{ mV}$ and $s(U_{0})=2.79\text{ mV}$ . The experimental standard deviation of the average value of 15 times measurement can be described as $s(\bar{U_{0}})=s(U_{0})/\sqrt{n}=2.79/\sqrt{15}=0.72\text{ mV}$ .

The standard uncertainty of the measurement repeatability of *U*_0_ was expressed as: u(U0¯)=s(U0¯) . So the relative standard uncertainty caused by *U*_0_ can be described as:

(12) $$u_{1rel}(S)=\frac{u(\bar{U_{0}})}{U_{0}}=\frac{0.72}{2144.32}=0.033\%,\;v_{1}(S)=14$$

The closed-loop nanopositioning stage (P-733.3CL) was used in the calibration of the optical lever sensitivity to generate a deflection of the cantilever. According to the performance test protocol provided by the manufacturer, the nanopositioning stage was calibrated by a laser interferometer (SP 120D, SIOS GmbH., Ilmenau, Germany). It is able to position 10 μm with 0.2 nm closed loop resolution in the Z direction. The nonlinearity and full range reproducibility of the stage were reported to be 2 nm and 1 nm.

The standard uncertainty component caused by instrument resolution, typical nonlinearity, and full range reproducibility of the stage were:

(13) $$u_{1}(D_{0})=\frac{0.2\text{ nm}}{2\sqrt{3}}=0.577\text{ nm},\;u_{2}(D_{0})=\frac{2\text{ nm}}{\sqrt{3}}=1.155\text{ nm},\;u_{3}(D_{0})=1\text{ nm}$$

The combined standard uncertainty of the components above was:

(14) $$u(D_{0})=\sqrt{u_{1}^{2}(D_{0})+u_{2}^{2}(D_{0})+u_{3}^{2}(D_{0})}=\sqrt{0.577^{2}+1.155^{2}+1^{2}}=1.529\text{ nm}$$

As the maximum displacement in the sensitivity calibration was $\bar{D_{0}}=1000\text{ nm}$ , the relative standard uncertainty and DOF of the displacement *D*_0_ were given by:

(15) $$u_{2rel}(S)=\frac{u(D_{0})}{\bar{D_{0}}}=\frac{1.529\text{nm}}{1000\text{nm}}=0.153\%,\;v_{2}(S)=\frac{1}{2\times {\left(10\%\right)}^{2}}=50$$

During the calibration, the laser spot should focus on a particular position of the cantilever. Any tiny movement of the focused laser spot will change the reflection angle and the optical lever sensitivity, so the laser spot shift on the cantilever *P* is an uncertainty component of the optical lever sensitivity *S*. *P* is defined as the ratio of the actual sensitivity *S*_2_ to the prior calibrated one *S_1_*, *P* = *S*_2_/*S*_1_. As the expectation of *P* is *P*_0_ = 1, the relative deviation of *P* is expressed in Equation (16). The detailed derivation will be discussed in the Appendix:

(16) $$\frac{P-P_{0}}{P_{0}}=\frac{S_{2}}{S_{1}}-1=\frac{\Delta x(2l-2x-\Delta x)}{2lx-x^{2}}$$

In Equation (16), *x* is the distance from the laser spot position to the fixed end of the cantilever; *l* is the distance from the tip to the fixed end of the cantilever; Δ*x* is the shift of the laser spot in longitudinal direction of the cantilever. Equation (16) decreases monotonically as *x* increases from 0 to *l*. According to our experimental experiences, the laser spot position ranges from *x* = 2*l*/3 to *x* = *l*. If we take the limit position *x* = 2*l*/3 to Equation (16), the maximum result of the equation could be expressed as:

(17) $${\left|\frac{P-P_{0}}{P_{0}}\right|}_{\text{max}}=\frac{6\Delta xl-9\Delta x^{2})}{8l^{2}}$$

For the rectangular cantilever (NSC15_F#1) discussed in this section, *l* was measured from the SEM images. The positioning error Δ*x* is smaller than 1 μm in our calibration system. If Δ*x* is assumed to be 1 μm, the maximum value of Equation (17) could be:

(18) $${\left|\frac{P-P_{0}}{P_{0}}\right|}_{\text{max}}=0.646\%$$

Based on a uniform distribution, the relative standard uncertainty component caused by laser spot shift on the cantilever was:

(19) $$u_{3rel}(S)=\frac{1}{\sqrt{3}}{\left|\frac{P-P_{0}}{P_{0}}\right|}_{\text{max}}=0.373\%,\;v_{3}(S)=\frac{1}{2\times {\left(20\%\right)}^{2}}=12.5$$

The combined relative standard uncertainty of the optical lever sensitivity *S* was contributed by the three uncertainty components mentioned above:

(20) $$u_{rel}(S)=\sqrt{u_{1rel}^{2}(S)+u_{2rel}^{2}(S)+u_{3rel}^{2}(S)}=\sqrt{0.033^{2}+0.153^{2}+0.373^{2}}=0.403\%$$

The effective DOF of *S* can be calculated by:

(21) $$v_{eff}(S)=\frac{u_{rel}^{4}(S)}{\sum_{i=1}^{3}\frac{u_{irel}^{4}(S)}{v_{i}(S)}}=16.9$$

### 4.5. Combined Uncertainty, u_crel_(k)

With the uncertainty components discussed above, the combined relative standard uncertainty of the calibrated spring constant can be described as:

(22) $$u_{crel}(k)=\sqrt{u_{rel}^{2}(m)+u_{rel}^{2}(g)+u_{rel}^{2}(S)}=\sqrt{0.038^{2}+0.010^{2}+0.403^{2}}=0.405\%$$

The calibration result and its standard uncertainty were given by:

(23) $$k=\frac{F}{x}=\frac{mgS}{U}=\frac{5.0042\times 9.8011\times 2144.32}{2000}=52.586\text{ N}\cdot m^{-1}$$

(24) $$u_{c}(k)=ku_{crel}(k)=52.586\times 0.405\%=0.213\text{ N}\cdot m^{-1}$$

The effective DOF of the combined standard uncertainty was:

(25) $$v_{eff}(k)=\frac{u_{crel}^{4}(k)}{\frac{u_{rel}^{4}(m)}{v_{eff}(m)}+\frac{u_{rel}^{4}(g)}{v_{eff}(g)}+\frac{u_{rel}^{4}(S)}{v_{eff}(S)}}=17.3$$

With the effective DOF, the coverage factor *k*_99_ = 2.895 (confidence probability *p* = 99%) was looked up from ***t*** distribution table. So the expanded uncertainty was calculated by:

(26) $$u_{99}(k)=k_{99}u_{c}(k)=2.895\times 0.213=0.617\text{ N}\cdot m^{-1}$$

Summarized in Table 3 are the uncertainty estimation results of the cantilever NSC15_F#1. We estimated the uncertainty of seven cantilevers calibrated with our method and summarized the estimation results in Table 4.

**Table 3:** Uncertainty estimation results of NSC15_F#1 cantilever.

Inputs	Expectation	Standard Uncertainty	Distribution	Relative Standard Uncertainty	DOF
***S***	**2144.32 mV·μm^−1^**	**8.65 mV·nm^−1^**	**Normal**	**0.403%**	**16.9**
*U*_0_	2144.32 mV	0.72 mV	Normal	0.033%	14
*D*_0_	1 μm	0.00153 μm	Normal	0.153%	50
*P*	1	----	Uniform	0.373%	12.5
***m***	**5.0042 mg**	**1.90 μg**	**Normal**	**0.038%**	**40.7**
Resolution	0	0.03 μg	Uniform	----	50
Reproducibility	0	0.25 μg	Uniform	----	50
Repeated measure	5.004 mg	1.88 μg	Normal	----	39
***g***	**9.8011 m·s^−2^**	**0.0010 m·s^−2^**	**Uniform**	**0.010%**	**50**
***U***	**2000 mV**	**----**	**----**	**----**	**----**
**Output (*k*)**	**52.586 N·m^−1^**	**0.213 N·m^−1^**	**Normal**	**0.405%**	**17.3**

*k*_99_ = 2.895 *U*_99_(*k*) = *k*_99_·*u*_c_(*k*) = 0.617 N·m^−1^. *k* = 52.586 ± 0.617 N·m^−1^.

**Table 4:** Uncertainty sources and the contribution to the combined uncertainty for seven cantilevers.

Uncertainty Source	Uncertainty Component	Relative Standard Uncertainty (%)						
		NSC11	CSG01	NSG01	MESP	NSC15_F		
						#1	#2	#3
**Added mass**	***U*_rel_(*m*)**	**0.115**	**2.500**	**0.074**	**0.092**	**0.038**	**0.057**	**0.077**
**Acceleration of gravity**	***U*_rel_(*g*)**	**0.010**	**0.010**	**0.010**	**0.010**	**0.010**	**0.010**	**0.010**
**Optical lever sensitivity**	***U*_rel_(*S*)**	**0.286**	**0.144**	**0.382**	**0.232**	**0.403**	**0.386**	**0.365**
PSD output signal	*U*_1rel_(*S*)	0.066	0.026	0.069	0.039	0.033	0.060	0.031
Nano stage displacement	*U*_2rel_(*S*)	0.153	0.051	0.153	0.102	0.153	0.153	0.153
Laser spot position shift	*U*_3rel_(*S*)	0.235	0.133	0.343	0.205	0.373	0.349	0.330
**Combined uncertainty**	***U*_crel_(*k*)**	**0.308**	**2.504**	**0.389**	**0.250**	**0.405**	**0.390**	**0.373**

The combined relative standard uncertainties of most cantilevers, except CSG01, were better than 1%. The uncertainty of the CSG01 cantilever was mainly a result of the uncertainty component of added mass. It could be greatly reduced by increasing the deflection of the cantilever, which will produce a larger added mass on the balance. However, a larger contact force between the tip and the balance may cause more damage to the silicon tip. In our experiment, the maximum deflection was limited to be within 3 μm. For other cantilevers, the uncertainty of laser spot position shift on the cantilever contributed most to the combined uncertainty, so in this calibration method, we must pay attention to any sources which may shift the laser spot position, and keep it constant during the calibration.

## 5. Results and Discussion

Table 5 lists the calibration results with the corresponding expanded uncertainty calculated in the same way as discussed in Section 4. The expanded relative standard uncertainties of the normal spring constant of most measured cantilevers (except CSG01) are smaller than 2%. Our calibration results of seven cantilevers fall into the distribution range of spring constant given by the manufacturer in Table 1. With the uncertainty estimation; we can get the effective normal spring constant for each measured cantilever with a credible uncertainty range. It makes the spring constant of the cantilever a precise and reliable value rather than an estimated nominal value with a large uncertainty range.

**Table 5:** Calibration resutls with expanded uncertainty.

Model	*k* (N·m^−1^)	*u*_crel_(*k*) (%)	*u*_c_(*k*) (N·m^−1^)	*v*_eff_(*k*)	*k*_99_	*U*_99_(*k*) (N·m^−1^)	*U*_99rel_(*k*) (%)	*k* (N·m^−1^)
**NSC11**	2.795	0.308	0.009	35.0	2.72	0.023	0.838	2.795 ± 0.023
**CSG01**	0.0273	2.504	0.0007	51.6	2.68	0.0018	6.711	0.0273 ± 0.0018
**NSG01**	8.316	0.389	0.032	24.9	2.79	0.090	1.085	8.316 ± 0.090
**MESP**	4.022	0.250	0.010	27.0	2.77	0.028	0.693	4.022 ± 0.028
**NSC15_F#1**	52.586	0.405	0.213	17.3	2.90	0.617	1.173	52.586 ± 0.617
**NSC15_F#2**	51.304	0.390	0.200	18.9	2.86	0.572	1.115	51.304 ± 0.572
**NSC15_F#3**	44.035	0.373	0.164	20.0	2.85	0.468	1.063	44.035 ± 0.468

In our balance method, the calibration principle is based on the basic definition of the spring constant (Hooke’s law). The bending force and the deflection of the cantilever were measured separately. Before a spring constant calibration, the balance is calibrated by running an internal calibration program provided by the manufacturer. A built-in standard weight is used to realize the traceability. The nanopositioning stage used in the measurement of optical lever sensitivity was calibrated by a laser interferometer. Hence, the experimental results of our balance method are traceable and accurate with high repeatability and small uncertainties. What’s more, our method can calibrate almost all kinds of cantilevers regardless of their shape and stiffness. Considering the advantages mentioned above, our method could be used as a reference standard to evaluate the accuracy of other methods [6].

Two kinds of dynamic methods (the Sader method and thermal tune method) were used to calibrate the cantilevers again. Different from our balance method, the spring constants given by dynamic methods are called intrinsic spring constants. If the cantilevers are used in AFM systems some correction factors must be applied to get the effective spring constant [28]. That increases the uncertainty in dynamic methods. The effective spring constants measured by different methods are listed in Table 6. The Sader method needs to measure the plan view dimensions of the cantilever accurately. It is a main uncertainty source in the calibration results. This method is typically used for rectangular cantilevers. For V-shaped cantilevers the dynamic response and spring constant of each kind of cantilever should be corrected individually [5], so the NSC11 cantilever was not calibrated with the Sader method. The calibration result of CSG01 cantilever in the Sader method is much larger than its typical range. That is because the resonant frequency measured by the instrument is not the fundamental resonant frequency which is required by the thermal tune method. The amplitude of its first order resonant frequency is very low. It’s hard for the instrument to collect the resonant response of the cantilever, so the measurement result of the CSG01 cantilever was discarded. The thermal tune method can calibrate most types of cantilevers regardless of their shape and dimensions. Because of the user-friendliness and easily practical realization, this method has been widely used in recent years. Table 6 also lists the relative deviation of other methods compared to our balance method. Stiffer cantilevers have larger relative deviations than that of the softer ones in the thermal tune method. As the thermal vibration amplitudes of stiffer cantilevers are quite small, it is hard to extract the thermal vibration signals which are submerged in the electrical and mechanical noises of the measurement system. This method is believed to achieve its best accuracy for cantilevers softer than 1 N·m^−1^.

**Table 6:** Comparison of calibration results with different methods.

Cantilevers	Effective Spring Constant Obtained from Various Methods (N·m^−1^)		
	Balance	Sader	Thermal Tune
**NSC11**	2.795	----	2.686 (−3.9%)
**CSG01**	0.0273	----	0.0276 (1.1%)
**NSG01**	8.316	7.761 (−6.7%)	7.882 (−5.2%)
**MESP**	4.022	4.103 (2.0%)	4.192 (4.2%)
**NSC15_F#1**	52.586	48.00 (−8.7%)	43.85 (−16.6%)
**NSC15_F#2**	51.304	49.07 (−4.4%)	40.13 (−21.8%)
**NSC15_F#3**	44.035	40.69 (−7.6%)	40.47 (−8.1%)

**Figure 5:**
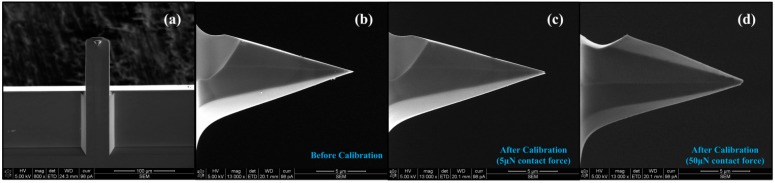
SEM images of a of NSC15_F probe before and after calibration. (**a**) is the bottom view of the cantilever; (**b**) is the side view of the tip before calibration; (**c**,**d**) are the side view of the tip after calibration.

Just as other static methods, damage may occur to the silicon tip apex in our method. In Section 3, the cantilever was bent 1 μm to evaluate the linearity of the calibration system. With the good linearity shown in Figure 3 and Figure 4, the maximum deflection of cantilever in the later calibration processes can be set to a much smaller value, e.g., 100~200 nm to decrease the contact force acts on the tip apex. Figure 5a,b show the SEM images of the cantilever and the tip of a new NSC15_F probe before calibration. Figure 5c,d show images of the damaged tip under different contact force of 5 μN and 50 μN (*i.e.*, different deflection). It is hard to avoid the tip apex damage, but smaller contact force (less than 5 μN) on the tip has no obvious influence on the resolution of AFM imaging in later usage, so it is suggested to perform the spring constant calibration after other measurement steps to avoid the influence of any possible tip damage.

## 6. Conclusions

In conclusion, we have introduced an AFM cantilever calibration method which could determine the normal spring constant with a small uncertainty. Our facility can directly measure the deflection and the bending force of the cantilever. Because of the closed-loop control and real-time measurement of the deflection, our balance method can avoid errors caused by the finite stiffness and thermal expansion of the measurement device, and keep the deflection of cantilever steady during the calibration. With this method, five kinds of commercial cantilevers with different shapes and nominal spring constants have been investigated in our study. Measurement and analysis of each cantilever indicates that our method is able to calibrate cantilevers with good repeatability and accuracy. In comparison with other methods, this method can be used as a reference standard in the spring constant calibration of AFM cantilevers and it could increase the reliability of force measurement applications that use AFM cantilevers as force sensors.

## Figures and Tables

**Figure A1:**
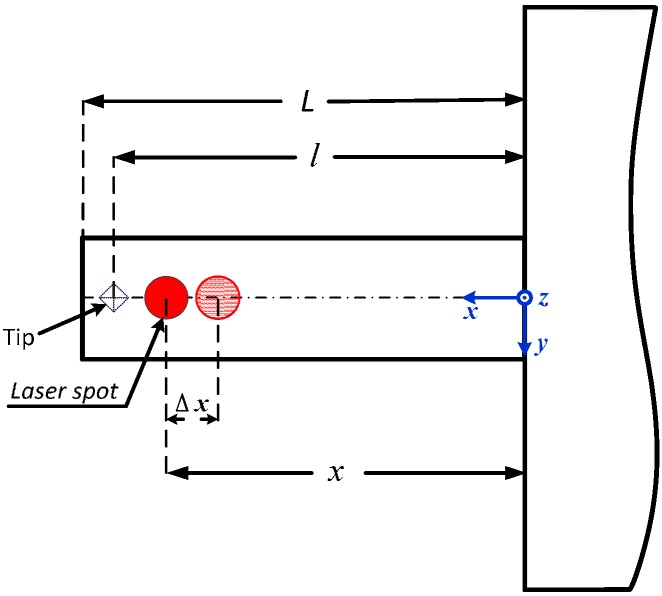
Schematic drawing of a rectangular beam model with laser spot on it.

## Appendix

A simple rectangular beam model was built to estimate the influence of the laser spot position shift on the cantilever to the optical lever sensitivity. If the cantilever beam can be simplified as a diving board with uniform cross section, it follows the Euler-Bernoulli beam theory. As shown in Figure A1, in order to simplify the calculation, the small inclination angle of the cantilever was not considered in our model. It has a neglected contribution to the estimation results. A normal force *F* (positive direction of Z-axis) is added to the tip of the cantilever, the displacement *h* of the rectangular beam in Z-axis at any position along the center line can be expressed as:

(A1)
$$h=-\frac{F}{6EI}x^{3}+\frac{Fl}{2EI}x^{2}$$

where *E* is the Young’s modulus of the cantilever, *I* is the sectional moment of the cantilever, *x* is the distance from the fixed end to the laser spot position, and *l* is the distance from the fixed end to the tip of the cantilever.

The deflection angle θ (in *x*-*z* plane) at laser spot position could be defined as:

(A2)
$$\theta \approx \text{tan }\theta =\frac{\partial h}{\partial x}=-\frac{F}{2EI}x^{2}+\frac{Fl}{EI}x$$

If the distances from the laser spot to the fixed end of the cantilever shifts from *x* to *x*-∆*x*, the deflection angle θ_1_, θ_2_ of the cantilever at different laser spot position can be expressed as:

(A3)
$$;\theta_{1}=-\frac{F}{2EI}x^{2}+\frac{Fl}{EI}x;\theta_{2}=-\frac{F}{2EI}{\left(x-\Delta x\right)}^{2}+\frac{Fl}{EI}(x-\Delta x)$$

Assuming the magnification of optical lever is *H*, the change of laser spot on the PSD is given by

(A4)
$$q_{1}=H\text{tan }2\theta_{1};q_{2}=H\text{tan }2\theta_{2}$$

As θ_1_ and θ_2_ are very small, then tan 2θ_1_≈ 2θ_1_, tan 2θ_2_≈ 2θ_2_, so Equation (A4) can be simplified as: *q*_1_≈ 2*H*θ_1_, *q*_2_≈ 2*H*θ_2_.

The shift of PSD output signals *U*_1_ and *U*_2_ are proportional to *q*_1_ and *q*_2_, and the scale factor is noted as *p*. If the displacement of the tip is δ, the optical lever sensitivity at different laser spot position can be described as follows:

(A5)
$$S_{1}=\frac{U_{1}}{\delta }=\frac{pq_{1}}{\delta };S_{2}=\frac{U_{2}}{\delta }=\frac{pq_{2}}{\delta }$$

If *P* = *S*_2_/*S*_1_, *P*_0_ = 1, then based on Equations (A3) and (A5),

(A6)
$$\frac{P-P_{0}}{P_{0}}=\frac{S_{2}}{S_{1}}-1=\frac{q_{2}-q_{1}}{q_{1}}=\frac{\theta_{2}-\theta_{1}}{\theta_{1}}=\frac{\Delta x(2l-2x-\Delta x)}{2lx-x^{2}}$$

In our calibration experiment, a kind of triangular shaped cantilever NSC11 was selected as an example. According to the parallel beam approximation (PBA) model, the two skewed rectangular arms of triangular shaped cantilever can be equivalent as two unskewed rectangular beam in parallel [8,29]. Then the analytical results for the rectangular cantilevers can be applied to triangular shaped cantilevers as well.
